# Drug Repurposing: Dipeptidyl Peptidase IV (DPP4) Inhibitors as Potential Agents to Treat SARS-CoV-2 (2019-nCoV) Infection

**DOI:** 10.3390/ph14010044

**Published:** 2021-01-08

**Authors:** Praveen P. N. Rao, Amy Trinh Pham, Arash Shakeri, Amna El Shatshat, Yusheng Zhao, Rahul C. Karuturi, Ahmed A. Hefny

**Affiliations:** School of Pharmacy, Health Sciences Campus, University of Waterloo, 200 University Avenue West, Waterloo, ON N2L 3G1, Canada; tn3pham@uwaterloo.ca (A.T.P.); a2shakeri@uwaterloo.ca (A.S.); arelshatshat@uwaterloo.ca (A.E.S.); yusheng.zhao@uwaterloo.ca (Y.Z.); rckaruturi@uwaterloo.ca (R.C.K.); ahmed.hefny@uwaterloo.ca (A.A.H.)

**Keywords:** drug repurposing, SARS-CoV-2 infection, dipeptidyl peptidase IV inhibitors, SARS-CoV-2 M^pro^ protomer, SARS-CoV-2 M^pro^ dimer, MERS-CoV CL^pro^, cysteine proteases, serine proteases, molecular docking, type-2 diabetes

## Abstract

The current outbreak of severe acute respiratory distress syndrome (SARS) or nCOVID-19 pandemic, caused by the coronavirus-2 (CoV-2), continues to wreak havoc globally. As novel vaccines are being discovered and developed, small molecule drugs still constitute a viable treatment option for SARS-CoV-2 infections due to their advantages such as superior patient compliance for oral therapies, reduced manufacturing costs and ease of large scale distribution due to better stability and storage profiles. Discovering new drugs for SARS-CoV-2 infections is a time consuming and expensive proposition. In this regard, drug repurposing is an appealing approach which can provide rapid access to therapeutics with proven record of safety and efficacy. We investigated the drug repurposing potential of a library of dipeptidyl peptidase 4 (DPP4) inhibitors which are currently marketed for type-2 diabetes as treatment option for SARS-CoV-2 infections. These computational studies led to the identification of three marketed DPP4 inhibitors; gemigliptin, linagliptin and evogliptin as potential inhibitors of SARS-CoV-2 M^pro^ viral cysteine protease. In addition, our computational modeling shows that these drugs have the potential to inhibit other viral cysteine proteases from the beta coronavirus family, including the SAR-CoV M^pro^ and MERS-CoV CL^pro^ suggesting their potential to be repurposed as broad-spectrum antiviral agents.

## 1. Introduction

The ongoing novel coronavirus infection or SARS-CoV-2 (COVID-19 or nCOVID-19), pandemic, has already claimed more than 1.6 million lives worldwide, and continues to spread across the world at a rapid pace [1]. Unfortunately, there are no effective therapies which can reduce its severity or cure this disease. Recent studies have shown that the anti-malarial drug hydroxychloroquine, the antibiotic azithromycin, antiparasitic drug ivermectin and the corticosteroid dexamethansone have the potential to reduce disease severity in patients with SARS-CoV-2 infection [2,3,4,5]. This preliminary evidence suggests that small molecule therapies hold promise in treating this global pandemic. This also highlights the fact that drug repurposing or the application of known marketed drugs, to treat novel diseases such as the current SARS-CoV-2 pandemic, is a practical approach that should be thoroughly investigated. Successful drug repurposing can identify safe and effective drugs to treat diseases in a short time span that can be rapidly deployed at short notice, instead of the need to spend 10–15 years typically required to discover and develop new drugs [6]. Drug repurposing approach provides billions of dollars in cost savings and can also dramatically reduce the time required to launch new drugs [7].

**Figure 1:**
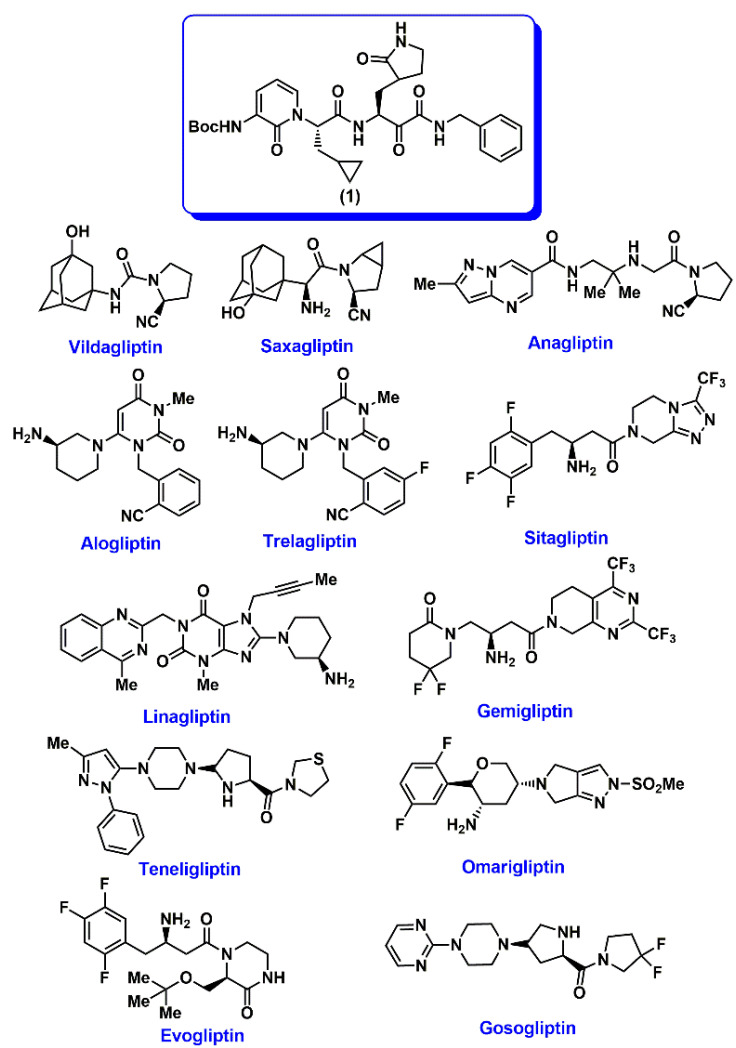
Chemical structures of SARS-CoV-2 M^pro^ inhibitor **1** and DPP4 inhibitors used in this study.

Recently, Zhang and coworkers made a seminal breakthrough in COVID-19 research by solving the crystal structure of SARS-CoV-2 viral protease, also called as main protease M^pro^ or 3CL^pro^ with a peptidomimetic α-ketoamide inhibitor (*tert*-butyl 1-((2*S*)-1-((2*S*)-4-(benzylamino)-3,4-dioxo-1-(2-oxopyrrolidin-3-yl)butan-2-ylamino)-3-cyclopropyl-1-oxopropan-2-yl)-2-oxo-1,2-dihydropyridin-3-ylcarbamate **1**, Figure 1) [8]. This study provided for the first time, structural insights into the M^pro^ cysteine protease, which is involved in the SARS-CoV-2 viral replication and is a desirable drug target as a treatment option to reduce and prevent SARS-CoV-2 infection in patients. The SARS-CoV-2 M^pro^ is a cysteine protease, and its catalytic site amino acids include His41 and Cys145 [8]. The M^pro^ dimer, is the catalytically active form. Inspired by this study, we started scanning the chemical structures of known FDA approved drugs for repurposing and were intrigued by dipeptidyl peptidase IV (DPP4 or CD26) inhibitors which are used in the treatment of type-2 diabetes [9,10,11,12]. The membrane bound DPP4 which is a serine protease, is active as a dimer and its catalytic site consists of Ser630, Asp708 and His740. Each monomer unit is made up of 760 amino acids [12,13]. Interestingly, human DPP4 was identified as the functional receptor for the human coronavirus-Erasmus Medical Center (hCoV-EMC) and Middle East Respiratory Syndrome Corona Virus (MERS-CoV) [14,15]. Furthermore, other studies revealed that DPP4 inhibition led to a reduction in the immunosuppressive effects of MERS-CoV infections [16,17]. Strikingly, the patient data from the COVID-19 outbreak in China has shown that patients with either type-1 or type-2 diabetes had greater mortality rate [18]. In particular, type-2 diabetes is the most common form of diabetes with a prevalence of >500 million cases worldwide [19] and these patients are at a greater risk of SARS-CoV-2 infections [20,21]. A recent study also demonstrated the effectiveness of DPP4 inhibitor sitagliptin in treating COVID-19 patients with type-2 diabetes although its mechanism of action in COVID-19 patients is not clear [22]. These facts convinced us to investigate the drug repurposing potential of FDA/market approved DPP4 inhibitors as treatment options for SARS-CoV-2 infections. In this regard, we studied the binding interactions of a library of 12 DPP4 inhibitors or gliptins—vildagliptin, saxagliptin, anagliptin, alogliptin, trelagliptin, sitagliptin, linagliptin, gemigliptin, tenegliptin, omarigliptin, evogliptin and gosogliptin (Figure 1) in the crystal structure of SARS-CoV-2 M^pro^ viral cysteine protease, by conducting molecular docking studies, pharmacophore modeling and by analyzing their molecular properties with the SARS-CoV-2 viral protease inhibitor **1** (Figure 1) [8]. Molecular docking studies were conducted in the binding sites of viral proteases of SARS-CoV-2, SARS-CoV, MERS-CoV and human DPP4 enzyme. These studies predict that DPP4 inhibitors, can exhibit good binding to SARS-CoV-2 viral protease suggesting their repurposing potential to treat SARS-CoV-2 infections in diabetic patients.

## 2. Results

### 2.1. Interaction of DPP4 Inhibitors in the SARS-CoV-2 M^pro^ Protomer

**Figure 2:**
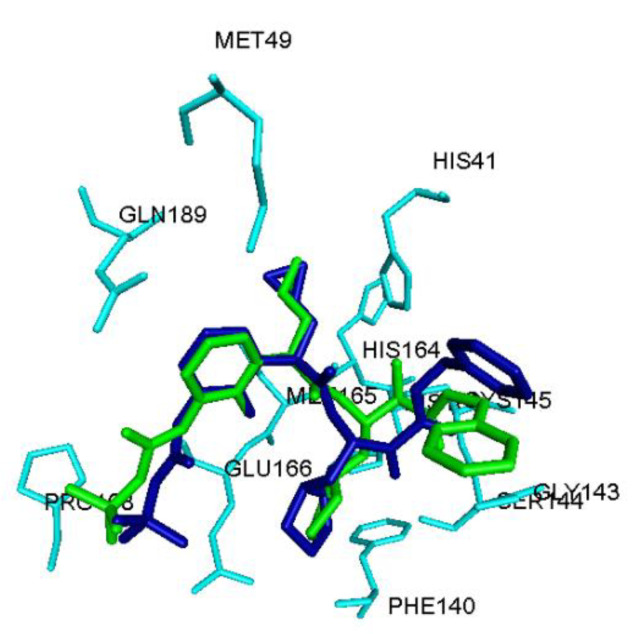
Comparison of binding mode of peptidomimetic inhibitor **1** (stick cartoon, blue color) in the SARS-CoV-2 M^pro^ protomer (PDB ID: 6Y2F) obtained using the CDOCKER docking algorithm with the crystal structure of **1** (stick cartoon, green color). Hydrogen atoms are not shown to enhance clarity.

The interaction of 12 DPP4 inhibitors (Figure 1) was investigated by conducting molecular docking studies using the crystal structure of SARS-CoV-2 M^pro^ protomer [8]. The viral cysteine protease protomer is made up of 306 amino acids and consists of three domains; domains I, II and III. The substrate binding site is present between domains I and II [8]. The molecular docking protocol was validated by docking the known SARS-CoV-2 M^pro^ inhibitor ((*tert*-butyl 1-((2*S*)-1-((2*S*)-4-(benzylamino)-3,4-dioxo-1-(2-oxopyrrolidin-3-yl)butan-2-ylamino)-3-cyclopropyl-1-oxopropan-2-yl)-2-oxo-1,2-dihydropyridin-3-yl-carbamate **1**), Figure 1) [8] using the CDOCKER alogorithm, after building **1** in 3D from scratch using the computational software Discovery Studio Structure-Based Design (BIOVIA, Dassault Systemes^®^, San Diego, CA, USA). This investigation shows that the peptidomimetic inhibitor **1**, exhibits a similar binding mode as per the crystallized inhibitor structure reported (all heavy atom RMSD = 1.70 Å, Figure 2) [8]. Consequently, molecular docking of 12 DPP4 inhibitors was carried out using CDOCKER algorithm. The binding modes were analyzed by ranking the best poses obtained using CDOCKER energies and CDOCKER interaction energies (Table 1).

**Figure 3:**
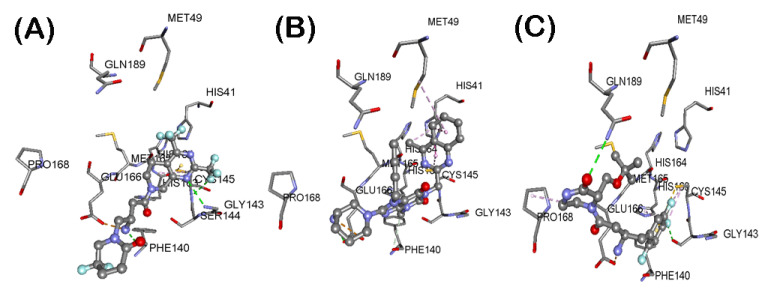
Binding modes of DPP4 inhibitors gemigliptin (**A**), linagliptin (**B**) and evogliptin (**C**) (stick cartoon) in the SARS-CoV-2 M^pro^ protomer (PDB ID: 6Y2F). Hydrogen atoms are not shown to enhance clarity.

These investigations identified three reversible, noncovalent DPP4 inhibitors—gemigliptin, linagliptin and evogliptin based on their superior CDOCKER energies which is a function of enzyme-ligand interaction energy (Table 1). Their ranking was of the order: gemigliptin (CDOCKER energy = −39.55 kcal/mol) > linagliptin (CDOCKER energy = −34.15 kcal/mol) > evogliptin (CDOCKER energy = −33.95 kcal/mol). Interestingly, these studies show that the dipeptide nitrile containing covalent DPP4 inhibitors vildagliptin, and saxagliptin formed high energy complex with the SARS-CoV-2 M^pro^ protomer which suggests that nitrile containing DPP4 inhibitors have the potential to undergo covalent binding with the catalytic site cysteine as reported by a previous study [23]. We investigated the binding modes of our top ranked reversible, noncovalent DPP4 inhibitors in the SAR-CoV-2 M^pro^ protomer (Figure 3). Gemigliptin was oriented in a linear conformation and the bicyclic dihydropyridopyrimidine ring was in the catalytic site and underwent hydrophobic interactions with catalytic site residues His41 and Cys145 (distance < 5.0 Å, Figure 3A). The C2 trifluoromethyl substituent underwent hydrogen bonding interactions with Gly143 and backbone NH of Cys145 (distance < 2.8 Å). The protonated primary amine, formed salt-bridge with side chain of Glu166 (distance = 1.79 Å). Interestingly, the difluoropiperidinone substituent was in a solvent exposed area and was forming an intramolecular hydrogen bonding interaction with one of the hydrogens of the pronated amine substituent, suggesting its role in reducing the flexibility across the C1-C2 single bond of oxobutyl spacer, linking the piperidine and dihydropyridopyrimidine rings (Figure 3A). Docking linagliptin in the SARS-CoV-2 protomer shows that the planar, bicyclic, 4-methylquinazoline ring was oriented toward the catalytic site in a perpendicular fashion with respect to the purine-dione ring and was in hydrophobic contact with His41, Met49 and Cys145 (distance < 5.0 Å, Figure 3B). The purine-dione ring was in contact with Gly143, Ser144 and His163 through polar and nonpolar interactions (distance < 5 Å) and the piperidine-amine substituent was closer to the entrance of the substrate binding region (Glu166, Leu167, Pro168 and Gln170). Similar to gemigliptin, the protonated primary amine was able to undergo salt-bridge with carboxylate side chain of Glu166 (distance = 4.4 Å, Figure 3B). Modeling DPP4 inhibitor evogliptin, shows that it exhibited a linear conformation such that the 2,4,5-trifluoromethylbenzene ring was oriented in the catalytic region (His41, Cyst145 and His163, Figure 3C) and the protonated primary amine formed a salt-bridge with carboxylate of Glu166 (distance < 2.90 Å). The methylpiperazinone substituent was closer to Leu167, Pro168 and Gln189. The C2 *tert*-butoxy substitutent was in van der Waal’s contact with side chain of Met49 (Figure 3C). These studies demonstrate that the amino acid residues in the S1 (Phe140, Leu141, Glu166 and Leu167) and S4 (Leu167, Pro168 and His172) pockets are flexible and can accommodate DPP4 inhibitors. Docking studies of other DPP4 inhibitors anagliptin, alogliptin, trelagliptin, sitagliptin, teneligliptin and gosogliptin, also shows their ability to interact in the substrate binding region of SARS-CoV-2 M^pro^ protomer, which shows their potential to act as inhibitors of viral protease (Figures S1 and S2, Supplementary Materials) although they were not as efficient, compared to gemigliptin, linagliptin and evogliptin based on their CDOCKER energies (Table 1).

### 2.2. Interaction of DPP4 Inhibitors Gemigliptin, Linagliptin and Evogliptin in the SARS-CoV-2 M^pro^ Dimer

**Figure 4:**
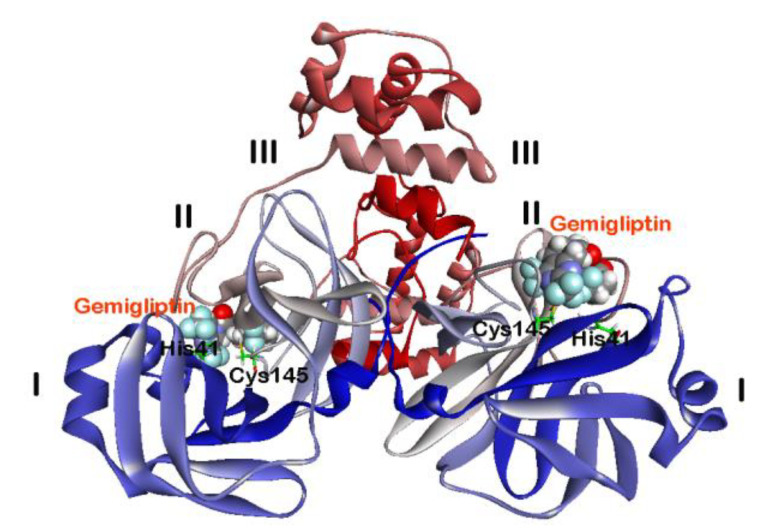
Binding modes of two molecules of DPP4 inhibitor gemigliptin (CPK cartoon) in the SARS-CoV-2 M^pro^ dimer (PDB ID: 6Y2G). Domains I–III are shown along with catalytic site amino acids His41 and Cys145. Hydrogen atoms are not shown to enhance clarity.

**Figure 5:**
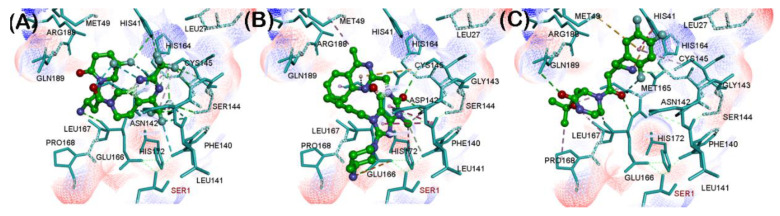
Binding modes of DPP4 inhibitors gemigliptin (**A**), linagliptin (**B**) and evogliptin (**C**) in the SARS-CoV-2 M^pro^ dimer (PDB ID: 6Y2G). The amino acid Ser1 from chain B is colored in red. Hydrogen atoms are not shown to enhance clarity.

The catalytically active form of SARS-CoV-2 M^pro^ is a dimer [8]. Therefore, we carried out molecular docking studies of DPP4 inhibitors using the crystal structure of SARS-CoV-2 M^pro^ dimer, to further understand their binding interactions with SARS-CoV-2 M^pro^. We selected DPP4 inhibitors gemigliptin, linagliptin and evogliptin in this study since they exhibited superior CDOCKER scores during our earlier docking investigation using the SARS-CoV-2 M^pro^ protomer (Figure 2 and Table 1). The docking protocol used was validated by carrying out molecular docking of compound **1** in the crystal structure of SARS-CoV-2 M^pro^ dimer which exhibited similar binding mode (RMSD = 1.83 Å) as per the crystal structure. Molecular docking studies on the whole SARS-CoV-2 M^pro^ dimer structure were conducted by sequentially docking DPP4 inhibitors at two substrate binding sites. As an example, interaction of two molecules of gemigliptin on the dimer is shown in Figure 4. In each protomers, the substrate binding site of SARS-CoV-2 M^pro^ is located at the surface of domains I and II. Interestingly, the shape of S1 pocket in the SARS-CoV-2 M^pro^ dimer is maintained by the interactions between Glu166 from chain A of one protomer, with Ser1 from chain B of another protomer [8]. The details of enzyme-ligand interaction of gemigliptin, linagliptin and evogliptin in the substrate binding site of SARS-CoV-2 M^pro^ is shown in Figure 5. As observed with the protomer docking studies, top ranked pose of gemigliptin exhibited superior CDOCKER energy (–32.62 kcal/mol) compared to linagliptin (CDOCKER energy = −28.90 kcal/mol) and evogliptin (CDOCKER energy = −28.50 kcal/mol) in the dimer. The known inhibitor **1**, exhibited superior binding (CDOCKER energy = −56.50 kcal/mol; CDOCKER interaction energy = −69.67 kcal/mol), which is expected, as it is a larger molecule that spans the entire substrate binding region of SARS-CoV-2 M^pro^ [8]. Molecular docking of gemigliptin shows that it was exhibiting a U-shaped conformation and was involved in several contacts in the SARS-CoV-2 M^pro^ substrate binding site (Figure 5A). The bicyclic dihydropyridopyrimidine ring was in the catalytic site surrounded by amino acids His41, Asn142, Gly143, Cys145 and His163 and the C4 trifluoromethyl substituent was in contact with the catalytic amino acids His41 and Cys145 via hydrogen bonding and hydrophobic interactions (distance < 5.0 Å). The C2 trifluoromethyl substituent underwent several hydrogen bonding interactions with Ser144 and His163 (distance < 3.0 Å) and was in contact with Cys145 (hydrophobic interactions, distance < 5 Å). The protonated primary amine formed a salt-bridge with Glu166 (distance = 1.79 Å) and the piperidine substituent was in a region consisting of Met49, Met165, Glu166, Leu167 and Gln189. The C5 difluoro-substituent of piperidinone was closer to Met165 side chain. These observations demonstrate that gemigliptin exhibits different binding modes in both protomer and dimer models of SARS-CoV-2 M^pro^ (RMSD = 3.83 Å). Next, we conducted docking studies of linagliptin in the SARS-CoV-2 M^pro^ dimer. It shows an L-shaped conformation in the catalytic site and the quinazoline substituent was in contact with Met49, Cys145 and Met165 (π-alkyl and π-sulfur interactions, distance < 5.0 Å, Figure 5B) whereas the purine-dione was closer to Leu141, Asn142, His163 and His172 and underwent both polar and nonpolar contacts (distance < 5.0 Å). As observed with gemigliptin, the protonated primary amine underwent salt-bridge/electrostatic interactions with Glu166 (distance < 5.0 Å). Its binding mode was similar to that observed in the SARS-CoV-2 M^pro^ protomer (RMSD = 1.94 Å, Figure 3B) and the only difference was in the orientation of piperidine substituent, which was closer to Leu167, Pro168 and Gln170 in the protomer binding (Figure 3B). This can be attributed to the flexibility in the S4 pocket region of SARS-CoV-2 M^pro^ dimer. Then, we investigated the binding interactions of evogliptin in the SARS-CoV-2 M^pro^ dimer. This DPP4 inhibitor was in a linear conformation with the 2,4,5-trifluoromethylbenzene ring oriented in the catalytic site (His41, Met49 and Cys145). The aromatic ring underwent π-π T-shaped interactions with His41 aromatic ring (distance < 5.0 Å), π-sulfur interaction with Met49 side chain (distance < 5.0 Å) and π-alkyl interaction with Cys145 (distance < 5.0 Å) as shown in Figure 5C. The protonated primary amine, underwent cation-π and hydrogen bonding interactions with His41 and His164 respectively and the butanone ketone underwent hydrogen bonding interactions with backbone NH of Glu166 (distance = 2.07 Å). The *tert*-butoxymethylpiperazinone substituent was oriented in a lipophilic region comprised of Met165, Leu167, Pro168 and Gln189 and the lipophilic *tert*-butoxy group was in van der Waal’s contact with Met165, Leu167 and Pro168 (distance < 5.0 Å). Evogliptin exhibits different contacts and binding mode (RMSD = 3.92 Å) when compared to its binding in the SARS-CoV-2 M^pro^ protomer (Figure 3C). These studies demonstrate that the substrate binding site in SARS-CoV-2 M^pro^ dimer is well defined due to the interactions of Ser1 from chain B at the N-terminus with Glu166 and Phe140 in the S1 pocket that helps to maintain the shape and activity of SARS-CoV-2 M^pro^ dimer [8]. This also explains the differences observed in the binding modes of gemigliptin, linagliptin and evogliptin observed during the docking studies conducted using either the SARS-CoV-2 M^pro^ protomer or dimer crystal structures. Lack of N-terminal Ser1 from chain B, can make the S1 and S4 regions of SARS-CoV-2 M^pro^ protomer, more flexible by exposing them to solvent; whereas presence of Ser1 in the dimer makes those regions less flexible and buries the Glu166 in the S1 pocket. Comparing the electrostatic potential energy surface of SARS-CoV-2 M^pro^ protomer and dimer substrate binding sites, clearly show these differences (Figure S3, Supplementary Materials).

Modeling the manually built known peptidomimetic inhibitor **1** (Figure 1) in the SARS-CoV-2 M^pro^ dimer, shows that it was exhibiting superior binding interactions (CDOCKER energy = −56.50 kcal/mol, CDOCKER interaction energy = −69.67 kcal/mol) compared to the top three DPP4 inhibitors from our study: gemigliptin (CDOCKER energy = −32.62 kcal/mol, CDOCKER interaction energy = −43.88 kcal/mol), linagliptin (CDOCKER energy = −28.90 kcal/mol, CDOCKER interaction energy = −45.34 kcal/mol) and evogliptin (CDOCKER energy = −28.51 kcal/mol, CDOCKER interaction energy = −37.07 kcal/mol), suggesting that these DPP4 inhibitors would exhibit reduced binding and inhibition of SARS-CoV-2 M^pro^ dimer compared to the peptidomimetic inhibitor **1**.

### 2.3. Interaction of DPP4 Inhibitors Gemigliptin, Linagliptin and Evogliptin in the SARS-CoV M^pro^ Dimer

**Figure 6:**
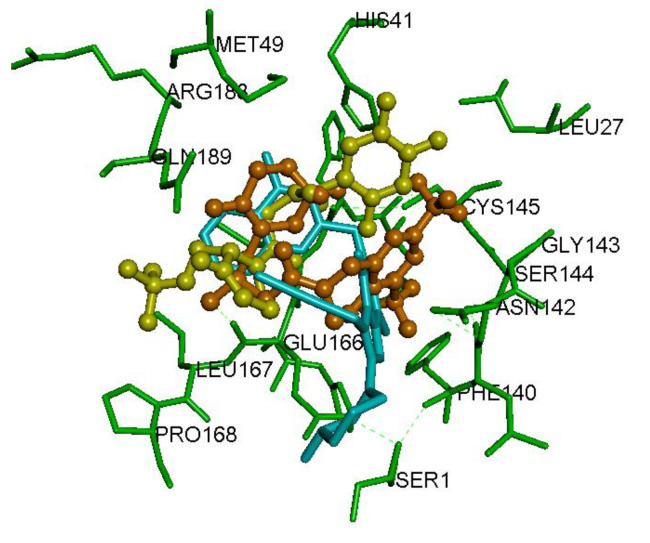
Binding modes of DPP4 inhibitors gemigliptin (orange stick cartoon), linagliptin (blue stick cartoon) and evogliptin (yellow stick cartoon) in the SARS-CoV M^pro^ dimer (PDB ID: 1UK4). Hydrogen atoms are not shown to enhance clarity.

We investigated the binding interactions of reversible, noncovalent DPP4 inhibitors gemigliptin, linagliptin and evogliptin in the active site of another coronavirus viral cysteine protease SAR-CoV M^pro^. This virus was responsible for the SARS outbreak in 2003 and has no FDA approved treatment till now [24,25]. The SAR-CoV M^pro^ shares 96% sequence identity with SARS-CoV-2 M^pro^ and is also a cysteine protease [8,26]. The dimer form of this viral cysteine protease is active. Similar to SARS-CoV-2 M^pro^ dimer, the catalytic site of SARS-CoV M^pro^ contains His41 and Cys145 and the N-terminal Ser1 from chain B maintains the shape of S1 pocket [26,27]. Molecular docking studies of gemigliptin shows that, it was binding in an extended conformation and was interacting in the entire span of SARS-CoV M^pro^ dimer binding site (Figure 6). The dihydropyridopyrimidine ring with trifluromethyl substituents was oriented in the catalytic site closer to His41, Met49, Asn142, Cys145 and Met165 and underwent hydrogen bonding (distance < 2.5 Å) and hydrophobic interactions (distance < 5.0 Å, Figure 6). The protonated amine was forming a salt-bridge (distance = 2.70 Å) with Glu166 carboxylate. The difluoropiperidinone was oriented toward a flexible region made up of Pro168 and Gln189 and were solvent exposed. Interaction of linagliptin in the SARS-CoV M^pro^ dimer binding site, shows that it exhibits an L-shaped conformation and the quinazoline ring was in contact with the catalytic site His41 (hydrogen bonding interaction, distance < 2.75 Å) and Cys145 (π-sulfur hydrophobic interactions). The planar purine-dione ring was closer to Met49, Leu167, Pro168 and Gln189 and the linear butynyl substituent was in van der Waal’s contact with Cys145 and aromatic ring of His163 (distance < 5.0 Å, Figure 6). The protonated amine of piperidine substituent, underwent salt-bridge with Glu166 carboxylate (distance = 4.78 Å). Molecular docking studies of evogliptin in the SARS-CoV M^pro^ dimer, shows that it adapts an S-shaped conformation with the trifluorobenzene substituent oriented closer to Leu167, Pro168 and Gln189, with one of the fluorines, undergoing polar interactions with Gln189 side chains (distance = 2.39 Å). Evogliptin exhibited weak interactions with catalytic site amino acid residues, except for the hydrophobic interactions of the Boc-substituent with the side chain of Cys145 (Figure 6). The piperazinone substituent was closer to Met49 and Met165 and the protonated amine formed salt-bridge with Glu166 carboxylate (distance = 3.52 Å) as observed with gemigliptin and linagliptin. The CDOCKER energies show that gemigliptin exhibited superior interactions in the SARS-CoV M^pro^ dimer compared to linagliptin and evogliptin. Their CDOCKER energies were of the order: gemigliptin (CDOCKER energy = −35.26 kcal/mol) > evogliptin (CDOCKER energy = −31.89 kcal/mol) ≈ linagliptin (CDOCKER energy = −31.81 kcal/mol). These results show that DPP4 inhibitors can bind to SARS-CoV viral protease.

### 2.4. Interaction of DPP4 Inhibitors Gemigliptin, Linagliptin and Evogliptin in the MERS-CoV CL^pro^ Dimer

The MERS-CoV virus is another contagious disease, which exhibited significantly greater mortality rate compared to SARS-CoV outbreak [28]. The X-ray crystal structure of MERS-CoV viral cysteine protease, MERS-CoV CL^pro^ has been solved. It shows 50% sequence identity with SARS-CoV protease [8,29,30,31,32,33,34]. The MERS-CoV CL^pro^ is a cysteine protease, and is made up of three domains similar to SARS-CoV M^pro^ and SARS-CoV-2 M^pro^ [30]. The dimer is the active form, whereas the monomer is inactive. The catalytic site contains Cys148 and His41 residues [30,34]. Docking the top three DPP4 inhibitors gemigliptin, linagliptin and evogliptin, based on our SARS-CoV M^pro^ and SARS-CoV-2 M^pro^ dimer modeling studies, shows that these three DPP4 inhibitors undergo efficient interactions in the MERS-CoV CL^pro^ dimer (Figure S4, Supplementary Materials). Gemigliptin exhibited a U-shaped conformation in the MERS-CoV CL^pro^ dimer binding site and the trifluromethyl substituted dihydropyridopyrimidine ring, underwent numerous polar and nonpolar contacts with His41, Ser147, Cys148 and His166 (distance < 5.0 Å). The piperidinone was closer to Met25, Leu27 and Cys145; whereas the protonated amine underwent hydrogen bonding interaction with His41 backbone (distance = 2.53 Å, Figure S4, Supplementary Materials). Linagliptin exhibited an L-shaped conformation, and the purine-dione aromatic ring was closer to the catalytic site and underwent π-π stacked, π-alkyl and alkyl-alkyl interactions with His41 and Leu49 (distance < 5.0 Å). The butynyl substituent, was oriented toward Cys148 and His166 and underwent alkyl-alkyl and π-alkyl interactions, respectively (distance < 5.0 Å). One of the purine-dione ketones, underwent hydrogen bonding interactions with Gln192 (distance = 2.48 Å) and the protonated amine group formed two hydrogen bonding interactions with Met25 and His41 (distance < 3.0 Å). A similar modeling of evogliptin in MERS-CoV CL^pro^ shows that, it exhibits an extended conformation and the trifluorobenzene substituent, was in van der Waal’s contact with Met168 and Gln192 (distance < 5.0 Å, Figure S4, Supplementary Materials). Interestingly, the Boc-substituent was oriented toward the catalytic site and was in van der Waal’s contact with His41 and Cys148 (distance < 5.0 Å). The CDOCKER energies obtained demonstrate that, gemigliptin was forming the most stable complex with MERS viral protease (CDOCKER energy = −38.55 kcal/mol), followed by linagliptin (CDOCKER energy = −31.65 kcal/mol) and evogliptin (CDOCKER energy = −31.60 kcal/mol). This study shows that DPP4 inhibitors have the potential to bind and inhibit MERS-CoV CL^pro^ dimer.

### 2.5. Pharmacophore Model of DPP4 Inhibitors toward SARS-CoV-2 M^pro^ Dimer

The top ranked poses obtained from the CDOCKER algorithm for gemigliptin, linagliptin and evogliptin were used to obtain pharmacophore model to identify the common structural features required for SARS-CoV-2 M^pro^ dimer binding. This study shows that the minimum structural feature requirements required, to bind in the substrate binding region of SARS-CoV-2 M^pro^ dimer, includes at least two hydrogen bond acceptors (HBA), two hydrophobic aliphatic (HPA) groups and at least one positively charged ionizable (POS) group, as shown in the pharmacophore model (Figure S5, Supplementary Information). This figure also provides distance parameters separating these chemical structure parameters and provides further insights on designing novel inhibitors of SARS-CoV-2 M^pro^ protease. The chemical structure (Figure 1) and binding mode of the DPP4 inhibitor gemigliptin in the SARS-CoV-2 M^pro^ dimer binding site (Figure 5) was analyzed. This shows that, the pyrimidine N2 and ketone substituent of piperidinone, act as HBA, with the C4 trifluoromethyl of the pyrimidine ring and aliphatic methylenes, which are part of the cyclic piperididone substituent, acting as hydrophobic aliphatic (HPA) groups, whereas the protonated amine group was acting as the POS group. This study also shows that the minimum structural requirements identified from the pharmacophore model, can interact in the S1, S2 and S3 pockets of SARS-CoV-2 M^pro^ dimer substrate binding site.

### 2.6. Physicochemical Properties of DPP4 Inhibitors

The 2D and 3D physicochemical properties of twelve DPP4 inhibitors and the reported SARS-CoV-2 M^pro^ inhibitor **1**, were determined to understand the significance of these parameters in SARS-CoV-2 M^pro^ inhibition and drug design. Parameters including molecular weights, the number of hydrogen bond acceptors, hydrogen bond donors, number of aromatic rings, number of rotatable bonds, polar surface area, molecular volume and AlogP values were calculated (Table S1, Supplementary Materials). These studies show that all the DPP4 inhibitors, obey Lipinski’s rule of five (RO5) such as possessing molecular weights below 500 Daltons, having less than 10-hydrogen bond acceptors, less than 5 hydrogen bond donors and log P values below 5 (Table S1, Supplementary Materials) [35]. The known inhibitor **1**, complied with all the rules, except for the molecular weight, which was slightly over (MW: 593.67, Table S1, Supplementary Materials). The DPP4 inhibitors gemigliptin, linagliptin and evogliptin which exhibited superior binding interactions in the SARS-CoV-2 M^pro^ protomer and dimer structures, exhibited molecular volumes in the range of 316–380 Å^3^. This shows that these molecules are smaller as compared to the known peptidomimetic SARS-CoV-2 M^pro^ inhibitor **1** (molecular volume = 474.71 Å^3^, Table S1, Supplementary Materials). This is along the expected lines as compound **1** is much larger and therefore is able to bind in the entire span of SARS-CoV-2 M^pro^ protomer (Figure 2) and dimer substrate binding sites [8]. Remarkably, the flexibility of ligands appears to play a significant role in their ability to bind in the SARS-CoV-2 M^pro^ dimer substrate binding site, as the known peptidomimetic inhibitor **1**, has fourteen C–C rotatable bonds. This is reflected by the fact that DPP4 inhibitors with superior binding interactions, contain at least 3 or more C–C rotatable bonds, with evogliptin containing 7-rotatable bonds (Table S1, Supplementary Materials), followed by linagliptin and anagliptin containing 6-each rotatable C–C bonds respectively.

## 3. Discussion

Novel SARS-CoV-2 outbreak is a global pandemic which has no definite cure as yet. In this regard, we investigated the drug repurposing strategy to shorten the time and cost required for the rapid deployment of known FDA approved/marketed drugs to treat SARS-CoV-2 infections. The current SARS-CoV-2 crisis has revealed that patients with pre-existing conditions such as type-2 diabetes have a greater risk of mortality due to SARS-CoV-2 infection and that the known DPP4 inhibitor sitagliptin was shown to be effective in treating COVID-19 infections in diabetic patients, although its exact mechanisms are not clearly understood [18,20,22,36]. Based on this evidence, we evaluated the drug repurposing potential of a class of reversible, noncovalent DPP4 inhibitors to bind and interact with SARS-CoV-2 M^pro^ the cysteine protease. Molecular docking studies of a library of 12 gliptin class of DPP4 inhibitors on the cysteine protease SARS-CoV-2 M^pro^ protomer, identified three reversible, noncovalent DPP4 inhibitors gemigliptin, linagliptin and evogliptin (Figure 3). Interestingly, these three DPP4 inhibitors were able to undergo favorable interactions in the substrate binding site and were in contact with catalytic site amino acids His41 and Cys148. Strikingly, all these three reversible, noncovalent DPP4 inhibitors contain a protonated amine substituent which underwent salt-bridge with the carboxylate side chain of Glu166, which is accessible in the S1 pocket. The SARS-CoV-2 M^pro^ protomer as such, is inactive and S1 and S4 sites are flexible. This is due to the lack of stabilizing effect exerted by the polar interactions of Ser1 from chain B, with Glu166 and Phe140 [8]. This was further confirmed by determining the electrostatic potential surfaces of crystal structures of SARS-CoV-2 M^pro^ protomer and SARS-CoV-2 M^pro^ dimer (Figure S3, Supplementary Materials), which clearly shows that Glu166, was more accessible in the SARS-CoV-2 M^pro^ protomer. This shows the flexibility of SARS-CoV-2 M^pro^ protomer, and suggests that it can accommodate reversible, noncovalent DPP4 inhibitors gemigliptin, lingagliptin and evogliptin. This study also shows that SARS-CoV-2 M^pro^ protomer docking can assist in developing novel dimerization inhibitors [27] of SARS-CoV-2 M^pro^ and that reversible, noncovalent DPP4 inhibitors have the potential to alter the global conformation of SARS-CoV-2 M^pro^ protomer, which has implications in modulating the dimerization process and activity of SARS-CoV-2 M^pro^ dimer.

The active form of SARS-CoV-2 M^pro^ is the dimer [8]. Therefore, we conducted molecular docking studies of reversible, noncovalent DPP4 inhibitors gemigliptin, linagliptin and evogliptin in the dimer crystal structure, to understand the ability of these drugs as inhibitors of SARS-CoV-2 M^pro^ dimer. These investigations show that DPP4 inhibitors, were able to exhibit favorable interactions, with gemigliptin forming the most stable complex (Figure 4). Interestingly, in the dimer structure, the S1 pocket is narrow with the Glu166 buried. This is due to the polar interactions of N-terminus Ser1 from chain B, with chain A amino acid residues Phe140 and Glu166, which maintains the shape of S1 pocket in the active SARS-CoV-2 M^pro^ dimer [8]. All the three reversible, noncovalent DPP4 inhibitors interacted with the catalytic site amino acid residues His41 and Cys145 and with other amino acids that line the substrate binding site including Glu166. Compared to SARS-CoV-2 M^pro^ protomer, the active dimer substrate binding site is narrow and smaller. Despite that, both gemigliptin and evogliptin were able to bind in the dimer. This is not surprising as both these DPP4 inhibitors are flexible and have 6 or more rotatable bonds (Table S1, Supplementary Materials). The accommodation of larger DPP4 inhibitor linagliptin (molecular volume = 380.38 Å^3^), was favored due to the flexibility of S3 (Gln189) and S4 (Pro168) pockets [8]. The DPP4 inhibitor evogliptin (Figure 1), which contains a bulky Boc group, was interacting in the flexible S3 and S4 pockets of SARS-CoV-2 M^pro^ dimer (Figure 5). Strikingly, the crystal structure of the known SARS-CoV-2 M^pro^ dimer inhibitor **1** (Figure 1) which also contains a Boc group, exhibited similar binding orientation where, the Boc group was in the flexible S3 and S4 pockets (Figure 2) [8]. This also suggests that the reversible, noncovalent DPP4 inhibitors gemigliptin, linagliptin and evogliptin are expected to exhibit reduced inhibition of SARS-CoV-2 M^pro^ compared to compound **1** due to their reduced molecular volume as they can interact in S1, S2 and S3 pockets, whereas due to its larger size (474.71 Å^3^), compound **1** can interact with S1, S2, S3 and S4 pockets in the SARS-CoV-2 M^pro^ dimer. To further understand the binding mechanisms, the known X-ray crystal structure of linagliptin bound to the serine protease DPP4 dimer was compared with the docked binding mode of linagliptin in the crystal structure of cysteine protease SARS-CoV-2 M^pro^ dimer [37,38] (Figure S6, Supplementary Materials). This shows some common interactions including the stabilization of the acidic glutamate with the protonated primary amine substituent of linagliptin in the both the enzymes and also the interaction of purine-dione substituent with the catalytic site Ser630 in DPP4 and Cys145 in SARS-CoV-2 M^pro^ suggesting that linagliptin has the potential to exhibit noncovalent inhibition in SARS-CoV-2 protease. (Figure S6, Supplementary Materials). These studies further support the ability of DPP4 inhibitors to inhibit SARS-CoV-2 M^pro^.

Previous research has shown that the peptidomimetic compound **1** (Figure 1), is a broad spectrum antiviral with activities against SARS-CoV M^pro^, SARS-CoV-2 M^pro^ and MERS-CoV CL^pro^ viral cysteine proteases via covalent interaction [8]. In this regard, we investigated the potential of reversible, noncovalent DPP4 inhibitors—gemigliptin, linagliptin and evogliptin to bind and inhibit the related betacoronas virus cysteine proteases, the SARS-CoV M^pro^ and MERS-CoV CL^pro^, which were responsible for the SARS and MERS outbreaks respectively [24,28,31]. Molecular docking studies of DPP4 inhibitors, in the SARS-CoV M^pro^ dimer shows that gemigliptin, linagliptin and evogliptin are able to undergo favorable interactions. In general, it should be noted that comparing the crystal structures of both SARS-CoV M^pro^ [26] and SARS-CoV-2 M^pro^ dimers [8], reveals some subtle differences in their conformation and interactions. For example, the S1 pocket in SARS-CoV-2 M^pro^ dimer is more compact and the Glu166 is buried, unlike the S1 pocket in SARS-CoV M^pro^ dimer where the Glu166 is more accessible. This could be due to the fact that the N-terminal Ser1 from chain B, in the SARS-CoV-2 M^pro^ dimer, undergoes at least 5-intramolecular polar interactions with four amino acids (Phe140, Asn142, Glu166 and His172), whereas in SARS-CoV M^pro^ dimer, Ser1 undergoes three polar interactions with three amino acids (Phe140, Glu166 and His172). Furthermore, in the SARS-CoV M^pro^ dimer, the S1 and S2 pockets are covered by a “lid” region which reduces the access of ligands to these regions [32]. Interestingly, in the SARS-CoV-2 M^pro^ dimer structure, both S1 and S2 regions are relatively more accessible which makes the overall binding site of SARS-CoV-2 M^pro^ larger, as compared to SARS-CoV M^pro^ dimer. These differences in their binding site regions, could be due to the formation of a tight dimer interface, in the SARS-CoV-2 M^pro^ [8]. This observation is further supported by the fact that the known peptidomimetic inhibitor **1** (Figure 1), with a larger molecular volume (474.71 Å^3^, Table S1, Supplementary Materials) and an extended conformation, was able to exhibit superior inhibition of SARS-CoV-2 M^pro^ (IC_50_ = 0.67 µM), compared to SARS-CoV M^pro^ (IC_50_ = 0.90 µM) [8]. Accordingly, while comparing the CDOCKER energies and CDOCKER interaction energies of DPP4 inhibitors gemilgliptin, linagliptin and evogliptin, we were able to notice that these drugs formed stable complexes with the SARS-CoV M^pro^ dimer and were able to undergo superior interactions compared to their interactions in the SARS-CoV-2 M^pro^ binding site. Interactions of gemigliptin, linagliptin and evogliptin in the MERS-CoV CL^pro^ dimer, shows that they exhibit even better interactions in the binding site of MERS-CoV CL^pro^ compared to either the SARS-CoV M^pro^ and SARS-CoV-2 M^pro^ viral proteases, based on the CDOCKER energy and CDOCKER interaction parameters. These studies, highlight the potential of DPP4 inhibitors as broad spectrum antiviral agents to treat betacoronavirus infections.

Previous studies have shown that DPP4 is a known target of MERS-CoV for host cell entry [14,39]. However, it is not the target receptor for SARS-CoV and the current SARS-CoV-2 virus [40]. Interestingly, DPP4 activity is increased in patients with type-2 diabetes and the increased risk of type-2 diabetic patients to SARS-CoV-2 infection, suggests that DPP4 class of drugs have the potential to be used as novel therapy [22,41]. Both gemigliptin and evogliptin are approved as drugs to treat type-2 diabetes patients in Republic of Korea and other countries, whereas linagliptin is approved to treat type-2 diabetes by US FDA, European Medicines Agency (EMA), China Food and Drug Administration (CFDA) and many other countries. Our studies suggest that repurposing DPP4 class of drugs, as treatment option for patients with SARS-CoV-2 (2019-nCov) infections should be thoroughly investigated, which can benefit a number of elderly patients suffering from comorbidities such as type-2 diabetes, who are more susceptible to SARS-CoV-2 infections.

## 4. Materials and Methods

### 4.1. Preparation of Ligands

The library of 12 DPP4 inhibitors (vildagliptin, saxagliptin, anagliptin, alogliptin, trelagliptin, sitagliptin, linagliptin, gemigliptin, tenegliptin, omarigliptin, evogliptin and gosogliptin) were either built from scratch or their coordinates were obtained from known X-ray structures. Except for gemigliptin and gosogliptin, all the other DPP4 inhibitor X-ray coordinates were obtained from RCSB protein data bank (rcsb.org), PDB ids: 6B1E, 3BJM, 3WQH, 3G0B, 5KBY, 4FFW, 2RGU, 3VJK, 4PNZ, 5Y7H. Gemigliptin, gosogliptin and the known SARS-CoV-2 inhibitor **1**, were initially built in 2D using ChemDraw Ultra 11, minimized using ChemDraw 3D Pro 11, saved as .sd files and were opened using Discovery Studio (DS) Client Structure-Based-Design software v17.1.0.1643 (BIOVIA Dassault Systemes^®^, San Diego, USA). All the DPP4 inhibitors and compound **1** were subjected to the *Prepare Ligands* command under *Small Molecule* module in DS which assigns all the hydrogens and charges at pH 7.4. After this, the ligands were subjected to energy minimization using 2000 steps of *Smart Minimizer* protocol (RMS gradient = 0.01 kcal/mol) using CHARMm force field and distance dependent dielectric constant.

### 4.2. Preparation of Target Proteases, the SARS-CoV-2 M^pro^ Protomer, SARS-CoV-2 M^pro^ Dimer, SARS-CoV M^pro^ Dimer, MERS-CoV CL^pro^ Dimer and DPP4 Dimer

The X-ray structure coordinates of SARS-CoV-2 M^pro^ protomer, SARS-CoV-2 M^pro^ dimer, SARS-CoV M^pro^ dimer, MERS-CoV CL^pro^ dimer and human DPP4 dimer, were obtained from RCSB protein data bank (rcsb.org). PDB ids: 6Y2F, 6Y2G, 1UK4, 4YLU and 2RGU were used to prepare these enzymes for molecular docking studies. The water molecules were deleted and all the enzymes were subjected to *Prepare Protein* command under the *Macromolecules* module in DS, which adds hydrogens and assigns force field (CHARMm) at pH 7.4. After that, the ligands bound to these enzymes were selected to create a 10 Å radius sphere, which was defined as the ligand binding site. The *Receptor-Ligand Interactions* module in DS, was used for this purpose. In the next step, the bound ligand was deleted and then the CHARMm force field was assigned to the enzyme/receptor assembly, using the *Simulation* module in DS.

### 4.3. Molecular Docking Studies of DPP4 Inhibitors with Viral Proteases

The molecular docking studies of prepared DPP4 inhibitors and compound **1** on prepared SARS-CoV-2 M^pro^ protomer, SARS-CoV-2 M^pro^ dimer, SARS-CoV M^pro^ dimer, MERS-CoV CL^pro^ dimer and human DPP4 dimer assemblies were carried out using the CDOCKER algorithm in the *Receptor-Ligand Interactions* module in DS using CHARMm force field [42]. CDOCKER algorithm uses simulated annealing protocol to determine the best ligand binding modes [43]. The protocol used 2000 heating steps, heating target temperature of 700 K, 5000 cooling steps and a cooling target temperature of 300 K. The binding modes of twelve DPP4 inhibitors in the substrate binding sites of SARS-CoV protease dimer, SARS-CoV-2 protease protomer/dimer and MERS-CoV viral protease were analyzed and ranked using the CDOCKER energy and CDOCKER interaction energy parameters. The CDOCKER energy score is based on the receptor-ligand interaction energy and the internal ligand strain energy whereas the CDOCKER interaction energy score indicates the nonbonded energy between the ligand and the protein/enzyme target. Greater energy scores indicate favorable ligand binding. Furthermore, various polar and nonpolar inter and intramolecular interactions, were analyzed to study the binding of DPP4 inhibitors with these enzymes. Root mean square deviation (RMSD) of binding orientation of DPP4 inhibitors, with various viral proteases were compared using the *Calculate RMSD* command under the *Small Molecules* module in DS, which calculates RMSD between various ligand binding modes by matching all the heavy atoms. Similar molecular docking studies were conducted using the known inhibitor **1** and its top binding mode obtained was compared with the known X-ray structure data [8].

### 4.4. Pharmacophore Modeling

The top ranked binding poses of DPP4 inhibitors gemigliptin, linagliptin and evogliptin, in the SARS-CoV-2 M^pro^ dimer substrate binding site, was used to generate a 3D-pharmacophore model using the *Common Feature Pharmacophore Generation* command under the *Pharmacophore* module in DS, which included parameters such as hydrogen bond acceptors (HBA), hydrogen bond donors (HBD), number of aromatic rings, hydrophobic aliphatic (HPA) substituents and polarizable charge (POS) groups. The distance parameters between individual chemical structure parameters were calculated in Angstrom (Å) units, using the *Measure* tool in DS.

### 4.5. Molecular Properties

The 3D physicochemical properties of all the DPP4 inhibitors used in this study were calculated using the *Molecular Properties* command under the *Small Molecules* module in DS. Parameters such as number of hydrogen bond acceptors/donors, polar surface area, molecular volume, number of rotatable bonds, number of aromatic rings and AlogP values for these ligands were calculated after subjecting the ligands to *Prepare Ligands* command at pH 7.4 and minimizing the ligands (2000 steps of *smart minimizer* protocol) using CHARMm force field. Molecular weights (MWs) reported are for the unprotonated structures of DPP4 inhibitors.

## 5. Conclusions

Our computational investigations have shown that the FDA approved DPP4 inhibitor linagliptin, along with two other DPP4 inhibitors gemigliptin and evogliptin which are marketed in the Republic of Korea to treat type-2 diabetes, have the potential to inhibit the SARS-CoV-2 M^pro^ viral cysteine protease by reversible, noncovalent binding suggesting their repurposing in treating COVID-19 infections. Furthermore, computational studies also indicate their potential in inhibiting SARS-CoV M^pro^ and MERS-CoV CL^pro^ viral cysteine proteases suggesting their application as broad-spectrum antiviral agents which warrants further investigations on their in vitro and in vivo activity evaluation to design and develop novel antiviral agents to target disease causing coronaviruses.

## Figures and Tables

**Table 1:** CDOCKER Energy and CDOCKER Interaction Energy data for DPP4 inhibitors in the SARS-CoV-2 M^pro^ protomer.

Compound Name	CDOCKER Energy in kcal/mol ^1^	CDOCKER Interaction Energy in kcal/mol ^1^
Anagliptin	−27.50	−45.29
Trelagliptin	−22.32	−46.04
Sitagliptin	−7.41	−40.13
Linagliptin	−34.15	−50.46
Gemigliptin	−39.55	−48.54
Tenegliptin	−16.74	−41.14
Evogliptin	−33.95	−39.96
Gosogliptin	−8.16	−37.98
**1**	−56.14	−70.00

^1^ The CDOCKER energy and CDOCKER interaction energies for the top ranked binding poses of DPP4 inhibitors obtained after conducting the molecular docking studies on the SARS-CoV-2 M^pro^ protomer (PDB ID: 6Y2F) using the CDOCKER algorithm in the software Discovery Studio Structure-Based-Design v17.1.0.1643 (BIOVIA Inc.).

## Data Availability

The data presented in this study is openly available in Research Square at https://www.researchsquare.com/article/rs-28134/v1 (DOI:10.21203/rs.3.rs-28134/v1) and also in the main text of this article and as Supplementary Materials.

## Supplementary Materials

The following are available online at https://www.mdpi.com/1424-8247/14/1/44/s1, Figure S1: Binding modes of DPP4 inhibitors anagliptin (A), alogliptin (B), trelagliptin (C) and sitagliptin (D) in the SARS-CoV-2 M^pro^ protomer. Figure S2: Binding modes of DPP4 inhibitors teneligliptin (A) and gosogliptin (B) in the SARS-CoV-2 M^pro^ protomer. Figure S3: Electrostatic surface potential map of SARS-CoV-2 M^pro^ protomer (A) and (B) dimer. Figure S4: Binding modes of DPP4 inhibitors gemigliptin, linagliptin and evogliptin in the MERS-CoV 3CL^pro^ dimer. Figure S5: Pharmacophore model to design SARS-CoV-2 M^pro^ dimer inhibitors based on the docked poses of DPP4 inhibitors - gemigliptin, linagliptin and evogliptin. Figure S6: 2D Interaction map of linagliptin in the active sites of the serine protease DPP4 and cysteine protease SARS-CoV-2 M^pro^. Table S1: Physicochemical properties of DPP4 inhibitors and the SARS-CoV-2 Mpro dimer inhibitor 1.
