# Supplementary material for: Drug Repurposing: Dipeptidyl Peptidase IV (DPP4) Inhibitors as Potential Agents to Treat SARS-CoV-2 (2019-nCoV) Infection
Source: Pharmaceuticals (Basel). 2021 Jan 8;14(1):44. doi: 10.3390/ph14010044 (PMC7827924; doi:10.3390/ph14010044)
Supplement: Supplementary file 1 [file pharmaceuticals-14-00044-s001.pdf]

Supporting Materials

Drug repurposing: Dipeptidyl peptidase IV (DPP4) inhibitors as potential agents to treat SARS-CoV-2 (2019-nCov) infection

Praveen P. N. Rao [1*], Amy Trinh Pham [1], Arash Shakeri [1], Amna El Shatshat [1], Yusheng Zhao [1], Rahul C. Karuturi [1] and Ahmed A. Hefny [1]

School of Pharmacy, University of Waterloo, Health Sciences Campus, 200 University Ave West, Waterloo, Ontario N2L 3G1, Canada

***Corresponding author**

Praveen P. N. Rao, School of Pharmacy, Health Sciences Campus, University of Waterloo, Waterloo, Ontario, Canada N2L 3G1, phone: 519-888-4567; ext: 21317; email: praopera@uwaterloo.ca

Contents

1. **Figure S1**: Binding modes of DPP4 inhibitors anagliptin (A), alogliptin (B), trelagliptin (C) and sitagliptin (D) in the SARS-CoV-2 M^{pro} protomer

2. **Figure S2**: Binding modes of DPP4 inhibitors teneligliptin (A) and gosogliptin (B) in the SARS-CoV-2 M^{pro} protomer

3. **Figure S3:** Electrostatic surface potential map of SARS-CoV-2 M^{pro} protomer (A) and (B) dimer

4. **Figure S4:** Binding modes of DPP4 inhibitors gemigliptin, linagliptin and evogliptin in the MERS-CoV $3CL^{pro}$ dimer

5. **Figure S5:** Pharmacophore model to design SARS-CoV-2 M^{pro} dimer inhibitors based on the docked poses of DPP4 inhibitors - gemigliptin, linagliptin and evogliptin

6. **Figure S6:** 2D Interaction map of linagliptin in the active sites of the serine protease DPP4 and cysteine protease SARS-CoV-2 M^{pro}

7. **Table S1:** Physicochemical properties of DPP4 inhibitors and the SARS-CoV-2 M^{pro} dimer inhibitor **1**

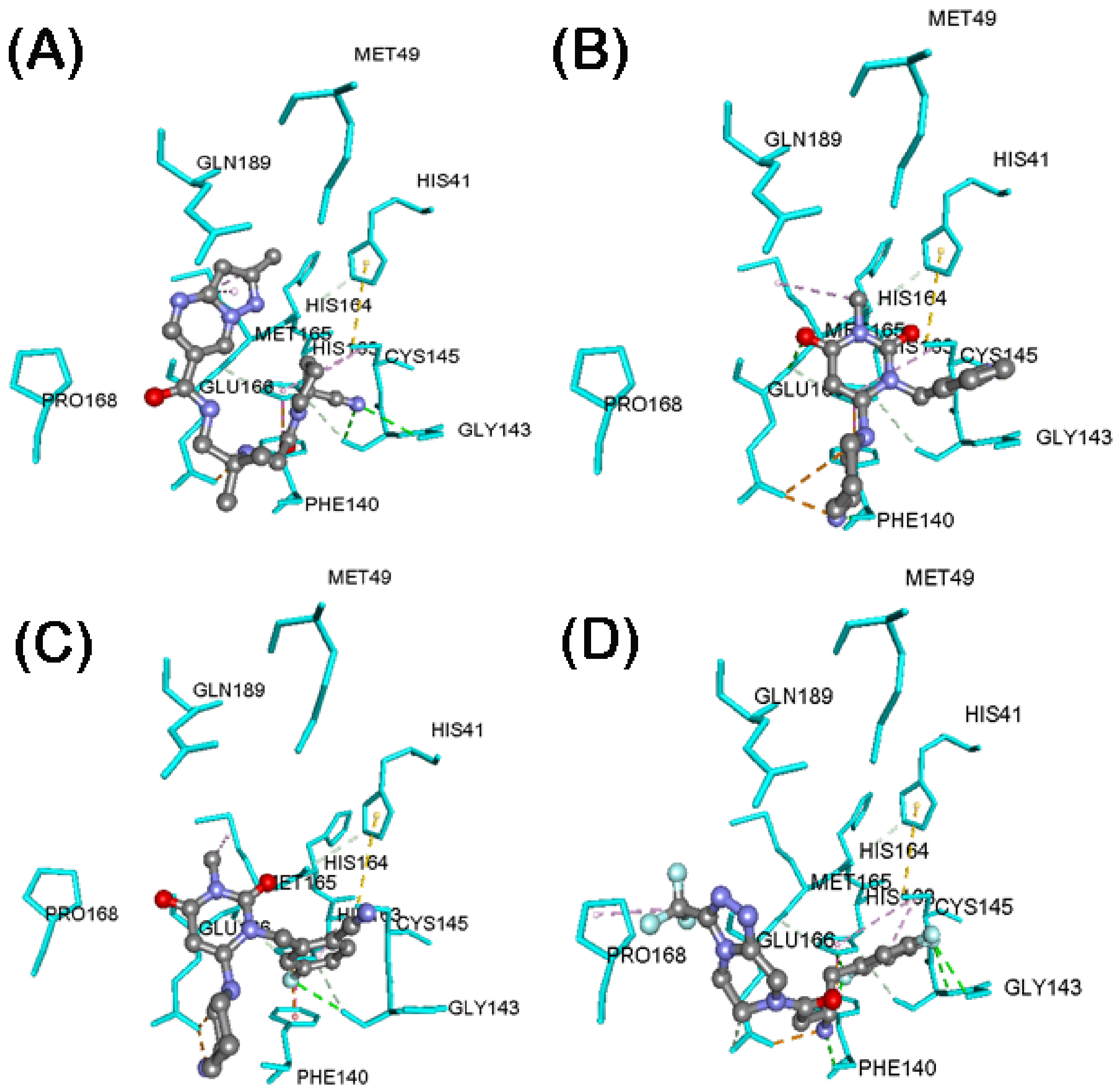

Figure S1. Binding modes of DPP4 inhibitors anagliptin (A), alogliptin (B), trelagliptin (C) and sitagliptin (D) in the SARS-CoV-2 M^{pro} protomer (PDB ID: 6Y2F). Hydrogen atoms are not shown to enhance clarity.

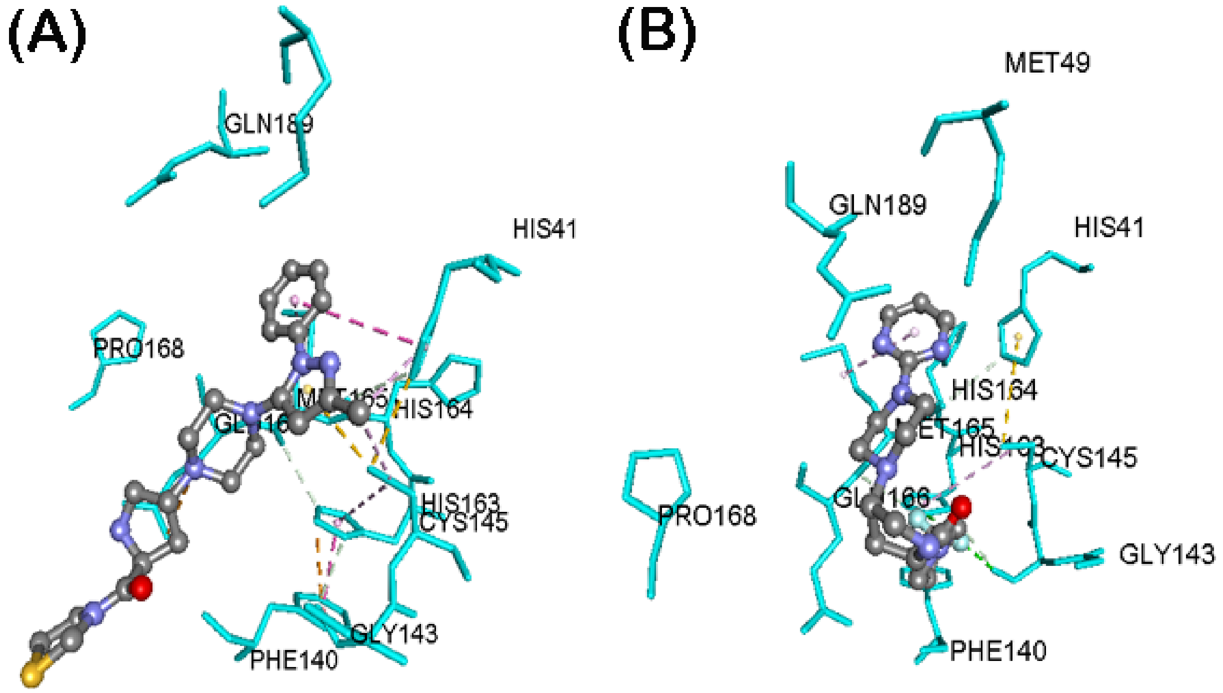

Figure S2. Binding modes of DPP4 inhibitors teneligliptin (A) and gosogliptin (B) in the SARS-CoV-2 M^{pro} protomer (PDB ID: 6Y2F). Hydrogen atoms are not shown to enhance clarity.

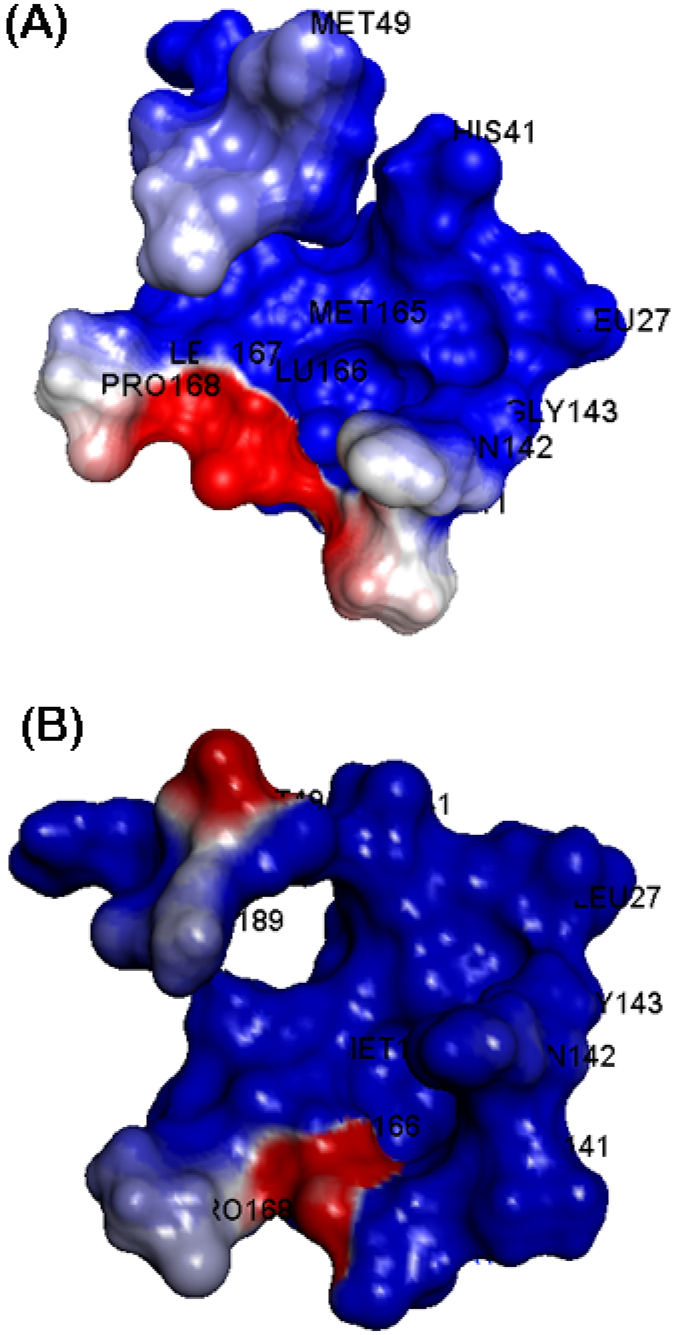

Figure S3. (A) Electrostatic surface potential map of SARS-CoV-2 M^{pro} protomer (PDB ID: 6Y2F) and (B) dimer (PDB ID: 6Y2G). Blue and red regions represent positive and negative surface respectively.

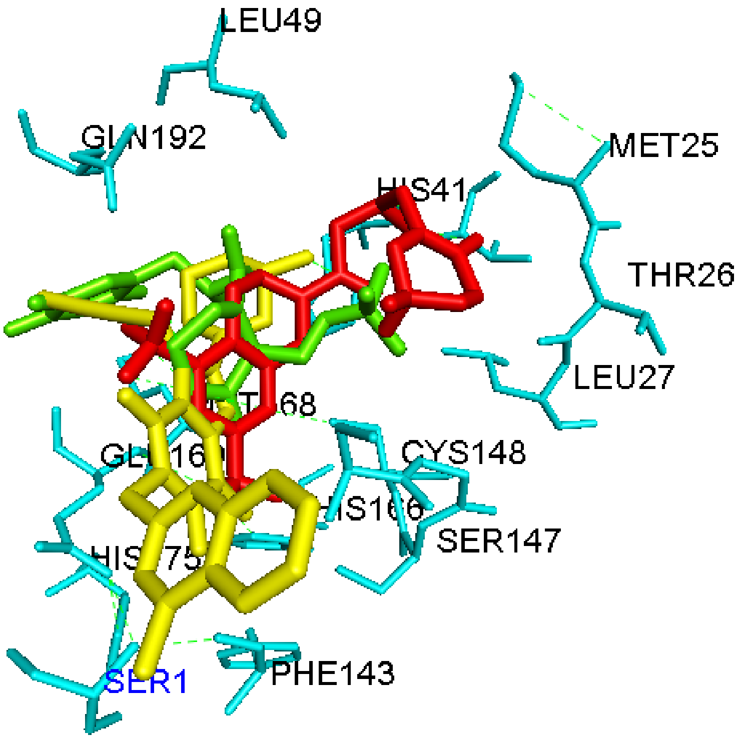

Figure S4. Binding modes of DPP4 inhibitors gemigliptin (red stick cartoon), linagliptin (yellow stick cartoon) and evogliptin (green stick cartoon) in the MERS-CoV 3CL^{pro} dimer (PDB ID: 4YLU). Hydrogen atoms are not shown to enhance clarity.

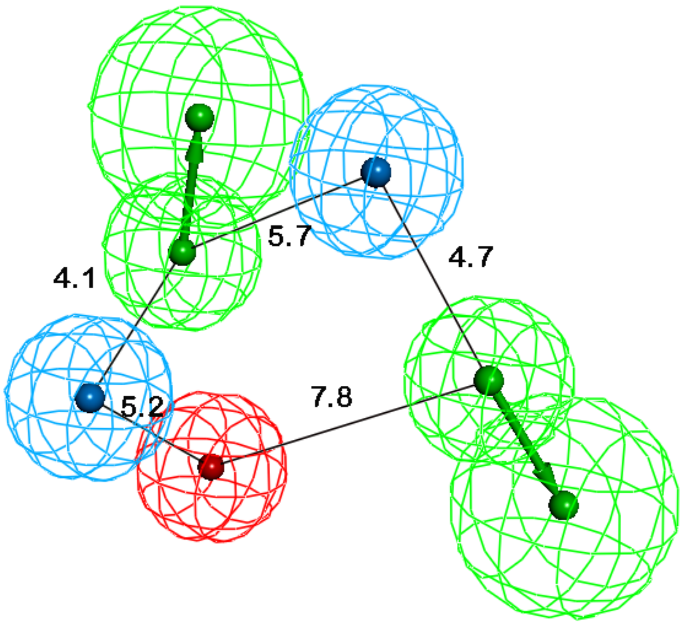

Figure S5. Pharmacophore model to design reversible, noncovalent SARS-CoV-2 M^{pro} dimer inhibitors based on the docked poses of DPP4 inhibitors - gemigliptin, linagliptin and evogliptin. Green contours represent hydrogen bond acceptors (HBA), blue contours represent hydrophobic aliphatic (HPA) groups and red contour represents polarizable charged (POS) group. Distance parameters are provided in Angstrom units (Å).

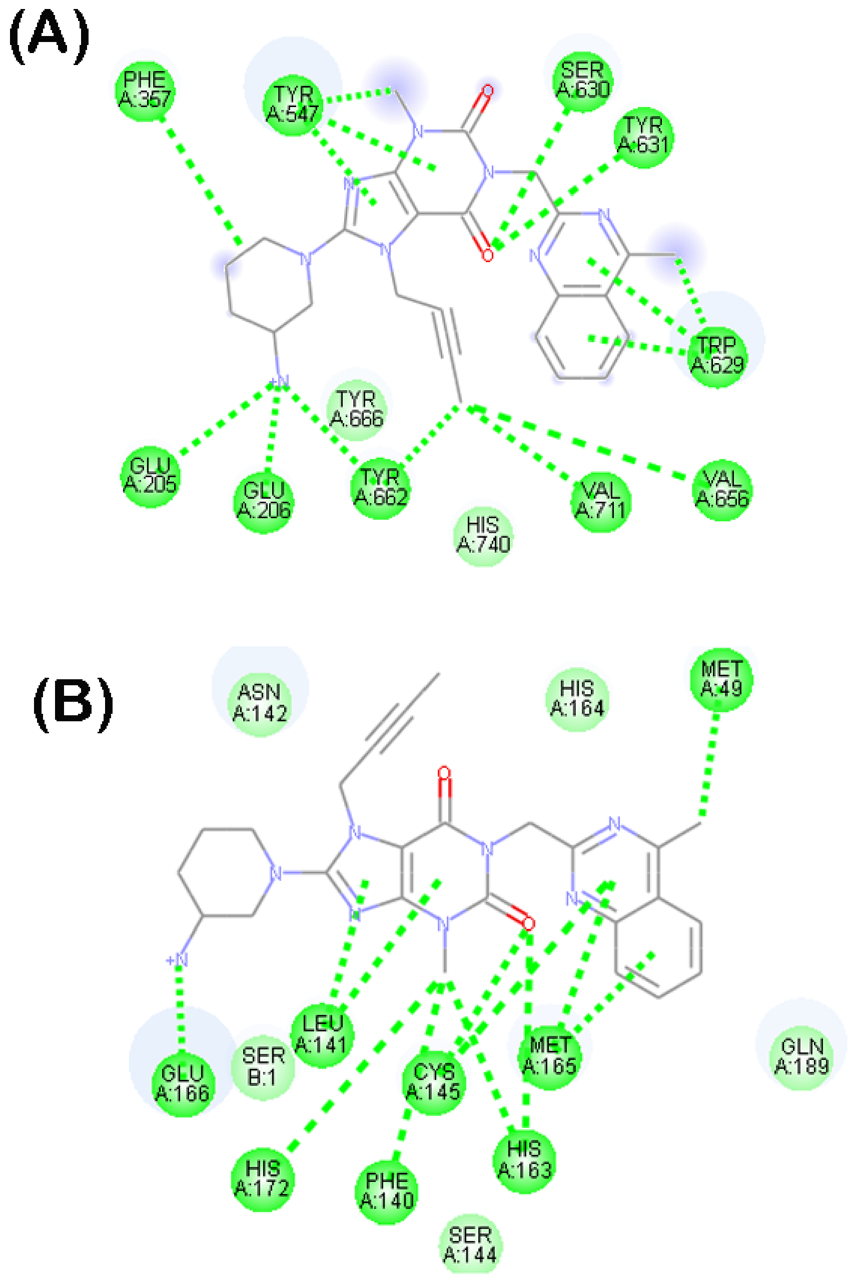

Figure S6. (A) 2D Interaction map of linagliptin in the active site of the serine protease DPP4 dimer (PDB ID: 2RGU). (B) 2D Interaction map of linagliptin in the active site of the cysteine protease SARS-CoV-2 M^{pro} dimer (PDB ID: 6Y2G). Hydrogen atoms are not shown to enhance clarity.

Table S1: Physicochemical properties of DPP4 inhibitors and the SARS-CoV-2 M^{pro} dimer inhibitor **1**

**Compound**	**No. of H-bond**[1]		**No. of rotatable bonds**[2]	**No. of aromatic rings**[3]	**Polar surface area (PSA in Å^2)**[4]	**Molecular weight**	**Molecular volume (Å^3)**[5]	**AlogP**[6]
	**Donors**	**Acceptors**						
Vildagliptin	2	3	3	0	0.257	289.37	254.16	–1.05
Saxagliptin	2	3	2	0	0.299	315.41	261.36	–0.73
Anagliptin	2	5	6	2	0.281	383.44	311.10	–1.85
Alogliptin	2	3	3	1	0.264	339.39	271.31	–1.29
Trelagliptin	2	3	3	1	0.257	357.38	283.66	–1.08
Sitagliptin	1	3	5	2	0.204	407.31	289.14	0.92
Linagliptin	1	6	5	4	0.230	472.54	380.38	1.71
Gemigliptin	1	4	6	1	0.204	489.36	336.82	0.28
Teneligliptin	2	5	4	2	0.191	426.57	350.54	0.35
Omarigliptin	2	4	3	2	0.266	398.42	300.12	–1.26

Evogliptin	2	3	7	1	0.200	401.42	316.93	0.225
Gosogliptin	1	5	3	1	0.186	366.40	282.63	–1.06
**1**	4	7	14	1	0.268	593.67	474.71	1.02

[1] Number of hydrogen bond donors/acceptors were calculated using *Discovery Studio Structure-Based-Design* (BIOVIA Inc) program using CHARMm force field. [2] Number of rotatable bonds, [3] Number of aromatic rings, [4] Polar surface area and [5] Molecular volume were calculated using the *Discovery Studio Structure-Based-Design* (BIOVIA Inc) program after energy minimization. [6] AlogP was calculated using CHARMm force field with the *Discovery Studio Structure-Based-Design* (BIOVIA Inc) program.
